# Unravelling the instability of mutational signatures extraction *via* archetypal analysis

**DOI:** 10.3389/fgene.2022.1049501

**Published:** 2023-01-04

**Authors:** Corrado Pancotti, Cesare Rollo, Giovanni Birolo, Silvia Benevenuta, Piero Fariselli, Tiziana Sanavia

**Affiliations:** Department of Medical Sciences, University of Torino, Torino, Italy

**Keywords:** archetypal analysis, mutational signatures, matrix factorization, COSMIC, cancer genomics

## Abstract

The high cosine similarity between some single-base substitution mutational signatures and their characteristic flat profiles could suggest the presence of overfitting and mathematical artefacts. The newest version (v3.3) of the signature database available in the Catalogue Of Somatic Mutations In *Cancer* (COSMIC) provides a collection of 79 mutational signatures, which has more than doubled with respect to previous version (30 profiles available in COSMIC signatures v2), making more critical the associations between signatures and specific mutagenic processes. This study both provides a systematic assessment of the *de novo* extraction task through simulation scenarios based on the latest version of the COSMIC signatures and highlights, through a novel approach using archetypal analysis, which COSMIC signatures are redundant and more likely to be considered as mathematical artefacts. 29 archetypes were able to reconstruct the profile of all the COSMIC signatures with cosine similarity > 0.8. Interestingly, these archetypes tend to group similar original signatures sharing either the same aetiology or similar biological processes. We believe that these findings will be useful to encourage the development of new *de novo* extraction methods avoiding the redundancy of information among the signatures while preserving the biological interpretation.

## Introduction

Somatic mutations in cancer are caused by a wide range of endogenous (i.e. genome instability or deficiency in a DNA repair mechanism) or exogenous (environmental exposures such as ultraviolet radiation or tobacco smoking) mutagenic agents, which stratify over time. It has been hypothesised that a mutational pattern in the genome can be deconvolved considering different generative processes, each of them associated with a specific mutational signature represented by 96 somatic mutation frequencies of six single nucleotide variants (*C* > *A*: *G* > *T*, *C* > *G*: *G* > *C*, *C* > *T*: *G* > *A*, *T* > *A*: *A* > *T*, *T* > *C*: *A* > *G*, *T* > *G*: *A* > *C*) flanked by one nucleotide on each side (Alexandrov et al., 2013). The latest version of the Catalogue Of Somatic Mutations In *Cancer* (COSMIC) (Bamford et al., 2004) hosts 79 single-base substitution (SBS) signatures extracted from 2,780 genomes of the *Pan Cancer* Analysis of Whole Genomes (PCAWG) (as described in https://cancer.sanger.ac.uk/signatures/sbs/) using SigProfilerExtractor (Islam et al., 2022), an updated version of the original method based on Non-Negative Matrix Factorization (NMF) proposed by Alexandrov et al. (2020).

Although signature extraction analysis is becoming a routine, there are some issues that should be further investigated. Recently, some studies have highlighted caveats and warnings in using these representations for clinical applications (Maura et al., 2019; Koh et al., 2021). Omichessan et al. (2019) did an empirical evaluation of the main *de novo* extraction tools showing that the identification of signatures is more difficult for tumours characterised by multiple signatures having a small contribution, and pointing out that different signatures might have very close cosine similarity, as it was observed between COSMIC signatures (cosine similarity > 0.8). Huang et al. used two complementary approaches to assess the confidence and stability of the resulting decomposition, showing that some mutagenic signatures (e.g., the signatures related to APOBEC activity) are more stable with respect to others, whose instability can be explained, in part, by the fact that these signatures can be decomposed into a linear combination of other signatures with a very small error (Huang et al., 2018). This was further tested by Schumann et al. (2019), who clearly showed that COSMIC signatures are, by construction, non-orthogonal, and this aspect affects the stability of the exposures. In particular, they computed the error between each COSMIC signature profile and its reconstruction using all the other signatures, finding four signatures with cosine similarity higher than 0.95 with respect to their reconstructed profile.

Lal et al. (2021) pointed out that state-of-the-art NMF-based methods aim to minimise the residual error after fitting the data with the discovered signatures to fit the data perfectly, which may generate overfitting issues by including stochastic noise in the data as part of the signatures, or multiple similar signatures for the same underlying process. Indeed, the goal of the signature discovery is not only to fit the data as well as possible, but also to identify signatures that truly reflect separate biological processes and the current version (3.3) of COSMIC database shows several signatures with no experimentally-validated aetiological causes associated yet, suggesting possible mathematical artefacts due to overfitting rather than distinct mutagenic processes (Koh et al., 2021). These issues become more critical when the signature extraction is highly dependent on the number of samples available, which complicates the correct identification of the true components, jeopardising the stability of the results. In addition, the studies of Maura et al. (2019) and Lal et al. (2021) highlighted that the presence of *flat signatures*, showing similar frequencies across all the 96 mutational classes, could represent a source of background noise and collinearities, making the *de novo* signature extraction task difficult and ambiguous.

**FIGURE 1:**
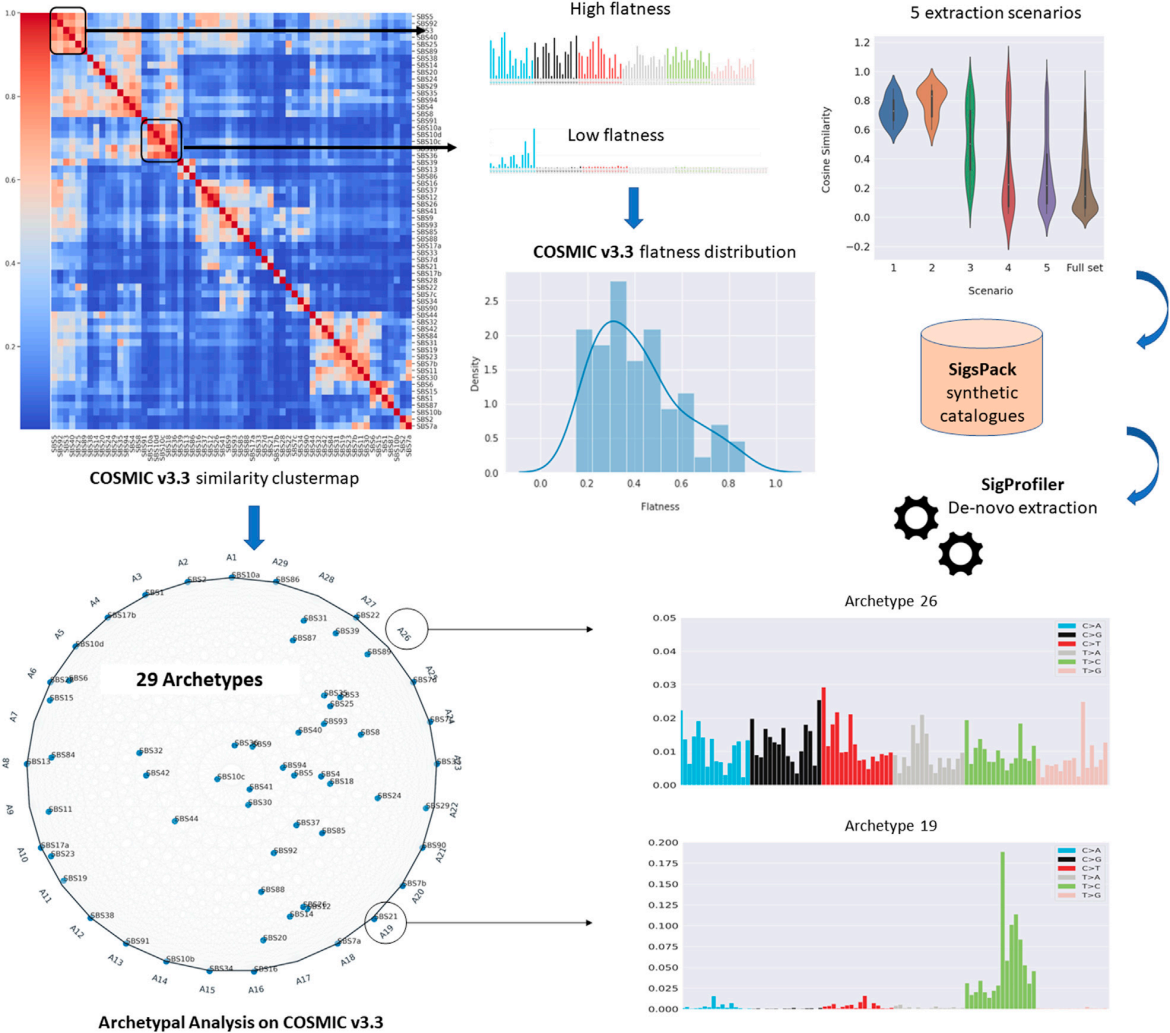
Summary of the analyses performed in this study. Firstly, we systematically assessed to which extent the extraction of the newest version of COSMIC signatures (v3.3) can be affected by the high similarity among the signatures in the same catalogue, the level of flatness of the signatures and the number of available samples, assessing the *de novo* extraction across five different scenarios (upper panel). Then, Archetypal Analyses was applied to provide a compact representation of the current catalogue by prioritising the identification of those profiles representing extreme patterns in the data so that all the observations can be reproduced as mixtures of their extremes (lower panel).

These issues are expected to become more critical in the newest version of COSMIC catalogue, which considers 79 signatures compared to the 30 signatures of the previous versions investigated in the above studies. Therefore, the proposed study will focus on two main goals: 1) to provide a systematic approach to assess to which extent the extraction of the newest version of COSMIC signatures can be affected by the high similarity among the signatures in the same catalogue, the presence of flat signatures and the number of available samples; 2) to provide a compact representation of the current catalogue by prioritising the identification of those profiles representing extreme patterns in the data so that all the observations can be reproduced as mixtures of their extremes. To this aim, Archetypal Analysis (Cutler and Breiman, 1994) was applied to represent how the information from COSMIC can be projected into a reduced number of dimensions and to explain potential instability issues in specific extraction scenarios. Figure 1 displays a summary of the workflow analysis proposed in this study.

## Materials and methods

### Similarity of COSMIC signatures

Analyses were performed on COSMIC catalogue v3.3 considering the 79 SBS mutational signature profiles on the reference genome GRCh37 identified by SigProfilerExtractor (Islam et al., 2022). Among these, we removed 19 signatures classified by the catalogue as sequencing artefacts (https://cancer.sanger.ac.uk/signatures/sbs/). Of the remaining 60 signatures considered, 19 neither have a direct association with an experimentally validated mutagenic process nor are they supported by statistical association with a specific process.

We first quantified the pairwise level of similarity in the signature catalogue. Therefore, for each pair of signatures **s**_**i**_ and **s**_**j**_ we calculated the cosine similarity:

$$cos\left(s_{i},s_{j}\right)=\frac{s_{i}\cdot s_{j}}{\left\|s_{i}\|\|s_{j}\right\|}$$ (1)

obtaining a pairwise cosine distance matrix $D\in R^{d\times d}$ , where *D*(*i*,*j*) = 1-sim(*i*,*j*) and *d* = 60 represents the number of signatures considered. We then built a cluster map to simulate different *de novo* extraction scenarios by applying to the *D* distance matrix a Hierarchical Clustering (Johnson, 1967) with average linkage.

### Flatness of COSMIC signatures

SBS3, SBS5, SBS40 and SBS8 are often referred to as flat signatures given their relative featureless profile, almost uniformly distributed across the 96 mutational classes. However, to the best of our knowledge, no quantitative definition of flatness has ever been provided. To fill this gap we formulated a simple definition of signature flatness by calculating the cosine similarity between the signature and the uniform distribution. Therefore the flatness of a signature **s**_**i**_ can be defined as:

$$flatness\left(s_{i}\right)=cos\left(s_{i},s_{u}\right)$$ (2)

Where **s**_**u**_, in the case of SBS mutational signatures, consists of a signal uniformly distributed over the 96 mutational classes. Hence, the flatness is a score ranging from 0 to 1, where 1 represents a perfectly flat profile. Since the presence of flat signatures in a catalogue can complicate the extraction task, a quantitative definition of flatness can be useful to build robust *de novo* extraction methods and to test their capabilities to correctly extract multiple signatures with different levels of flatness. In this regard, we designed scenarios with different levels of similarity and flatness.

### *De novo* extraction scenarios

To assess both the reliability and the feasibility of the *de novo* extraction procedure, several synthetic catalogues were generated considering COSMIC mutational signatures as underlying generative processes. The function *create_mut_catalogues* from SigsPack R package (https://github.com/bihealth/SigsPack) was used to generate 10 synthetic mutational catalogues for each extraction scenario to take into account statistical fluctuations (Schumann et al., 2019). In particular, *create_mut_catalogues* takes *samples*, *mutations* (per sample) and *signatures* as input and it generates mutational catalogues with exposures to the specified signatures by sampling the mutations from a distribution of those signature profiles. The contribution of each signature (exposure) is randomly drawn from a uniform distribution for each sample. Different ranges between a minimum of 200 and a maximum of 10,000 samples were set according to the chosen scenario and the number of signatures considered. The number of mutations in each tumour sample was set to 5,000 for each scenario, based on the PCAWG median number of sample mutations. All the simulated scenarios are summarised in Table 1 and the generated catalogues are available at the Github repository https://github.com/compbiomed-unito/archetypal-analysis-cosmic.

**TABLE 1:** Summary of the *de novo* extraction scenario. For each simulated scenario, the number of active signatures, the cosine similarity level. the flatness level and the n° of samples.

Scenario	No of latent Signatures	Median Similarity	Median Flatness	No of Samples
1	6	0.73	0.76	200,500
2	5	0.83	0.34	200,500
3	11	0.50	0.64	200,500,1000,3000,5000,10000
4	11	0.22	0.44	200,500,1000,3000,5000
5	20	0.22	0.45	1000,3000,5000,10000

*De novo* extraction analysis was applied to each scenario using the gold-standard approach SigProfilerExtractor (Islam et al., 2022) (https://github.com/AlexandrovLab/SigProfiler), with the aim of evaluating the extraction performance from catalogues with groups of similar latent signatures, varying in number and flatness score. To choose the optimal number of latent signatures, SigProfilerExtractor performs a repeated NMF for a range of *k* operative signatures. For each value of *k*, this algorithm applies a custom partition clustering based on the Hungarian algorithm to the signature matrices resulting from the repeated NMF, so that a final consensus signature matrix is obtained. The number of NMF repetitions can be specified by the user. In our experiments this value was set to 30. All the other default parameters were used. To efficiently run SigProfilerExtractor, computational resources from HPC4AI center (Aldinucci et al., 2018) and Occam cluster (Aldinucci et al., 2017) were used, for a total of 64 CPUs and 6 GPUs.

### Evaluation metrics

Four metrics were considered for the performance evaluations:

• Frequency (*F*) of simulation runs where all the signatures are correctly identified:

$$F=\frac{\text{N° of successful runs}}{\text{N° of total runs}}$$ (3)

• Mean square error (MSE) between simulated and reconstructed catalogues:

$$MSE=\frac{\sum_{i=1}^{n}\sum_{j=1}^{m}{\left(x_{i,j}-{\hat{x}}_{i,j}\right)}^{2}}{n\cdot m}$$ (4)

where *x*_*i*,*j*_ and ${\hat{x}}_{i,j}$ are the matrix elements of the original $X\in R^{n\times m}$ and the reconstructed $\hat{X}\in R^{n\times m}$ catalogues, respectively.

• Average stability (*C*_*mean*_) measured by the mean silhouette coefficient score of the signature clusters generated by SigProfilerExtractor:

$$C_{\mathrm{mean}}=\frac{\sum_{k=1}^{K}\sum_{i=1}^{N}c_{ik}}{K\cdot N}=\frac{\sum_{k=1}^{K}C_{k}}{K}$$ (5)

*c*_*ik*_ is the silhouette coefficient of the *i*−*th* sample which belongs to cluster k. *N* is the number of NMF runs performed by SigProfilerExtractor, *K* is the number of cluster labels, and *C*_*k*_ is the mean silhouette score of the *k*−*th* cluster.

*c*_*ik*_ is given by:

$$c_{ik}=\frac{b_{ik}-a_{ik}}{max\left(a_{ik},b_{ik}\right)}$$ (6)

where *a*_*ik*_ is the mean intra-cluster distance and *b*_*ik*_ is the mean nearest-cluster distance of the *i-th* sample which belongs to cluster k.

• Minimum stability (*C*_*min*_), represented by the minimum silhouette coefficient score of the signature clusters generated by SigProfilerExtractor:

$$C_{min}=min\left\{C_{k}\right\},\;\;with\;\;k=1,\ldots ,K$$ (7)

### Archetypal analysis

Archetypal analysis (AA) is an unsupervised learning method that aims to represent data points as sparse convex combinations of their extreme elements. More formally, let $X\in R^{p\times q}$ be a matrix whose row vectors are $x_{i}\;\in R^{q}$ and let $Z\in R^{r\times q}$ be another matrix, whose column vectors **z**_**k**_ ∈ **R** ^*q*^ represent the archetypes. AA reconstructs *X* through a linear combination of archetypes **z**_**k**_, which are themselves convex combinations of the *X* rows **x**_**i**_. Therefore, AA solves the following constrained equation:

$$minimize\;\overset{p}{\underset{i=1}{\sum }}\|x_{i}-\overset{r}{\underset{k=1}{\sum }}\alpha_{ik}z_{k}{\|}^{2}\;with\;z_{k}=\overset{q}{\underset{j=1}{\sum }}\beta_{kj}x_{j}$$ (8)

where *α*_*ik*_ ≥ 0; $\sum_{k=1}^{r}\alpha_{ik}=1$ and *β*_*kj*_ ≥ 0; $\sum_{j=1}^{q}\beta_{kj}=1$ ensure that the archetypes fall on the convex hull of *X*.

In this study, AA was applied directly to the COSMIC signature matrix $M\in R^{60\times 96}$ where, as reported above, 60 is the number of COSMIC SBS mutational signatures and 96 are the mutation contexts. Since AA is a matrix decomposition technique, the number of basis elements has to be chosen, which in our case corresponds to the number of archetypes. This number was set in order to explain the 95% of the variance. AA was performed using the Python-based *Archetypal Analysis Package* freely available at https://data.csiro.au/collection/csiro:40600v1 (Motevalli Soumehsaraei and Barnard, 2019). The code and the archetypal profiles are available at the Github repository https://github.com/compbiomed-unito/archetypal-analysis-cosmic.

## Results

### COSMIC cluster map

**FIGURE 2:**
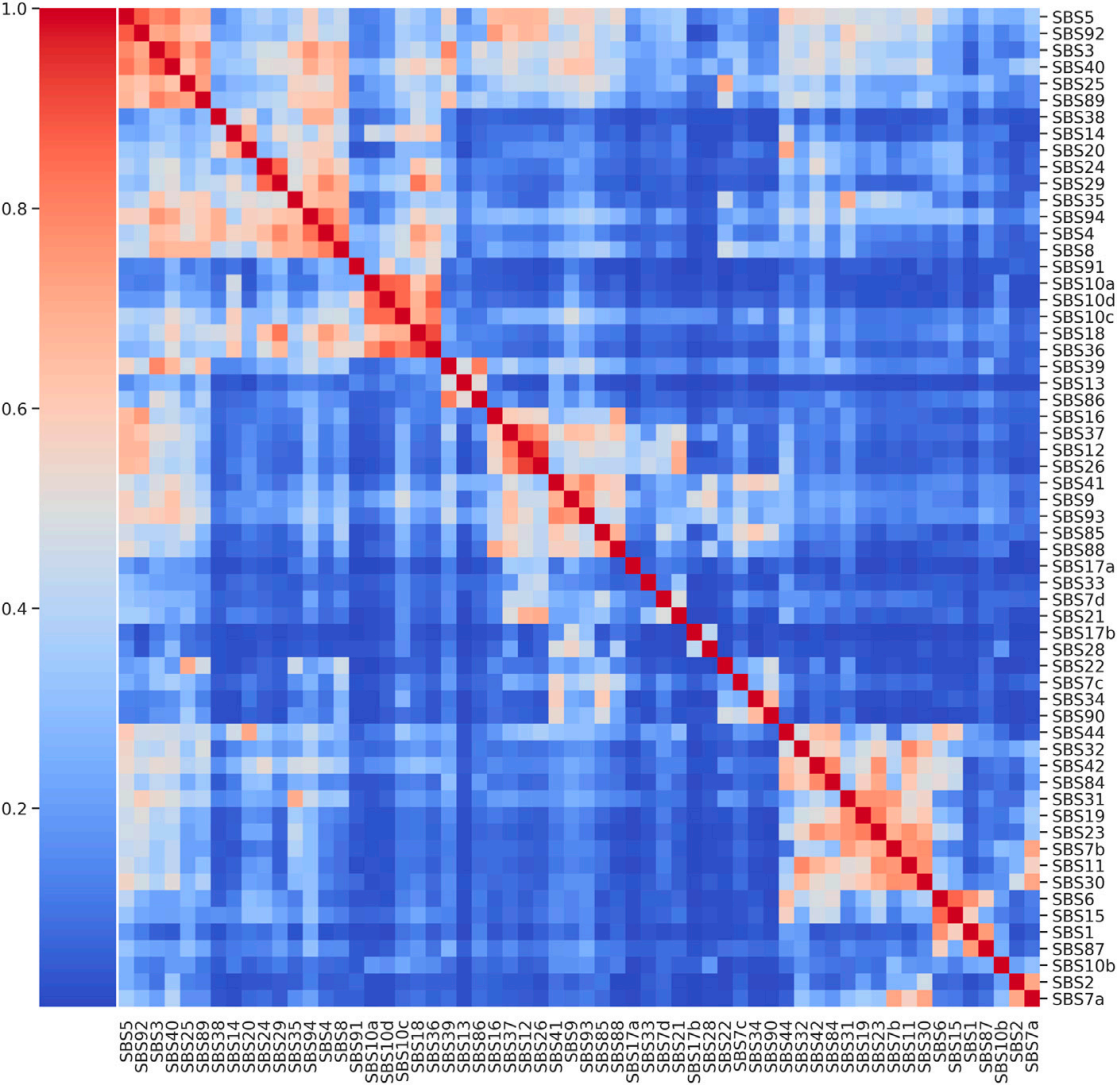
Cluster map of COSMIC SBS Mutational Signatures. Pairwise cosine similarity displayed for the 60 SBS signatures from COSMIC catalogue.

The cluster map on COSMIC v3.3 catalogue revealed that there are several groups of signatures showing pairwise cosine similarity >0.8 (Figure 2). The first group in the top-left corner consists of six signatures and it includes those signatures which are commonly referred to as *flat*, i.e. presenting a relatively featureless profile distributed over all the 96 mutational contexts, as the well-known SBS3 experimentally associated with defective homologous recombination-based DNA damage repair and the clock-like SBS5, statistically associated with age. The median pairwise similarity is 0.73, with a maximum equal to 0.88 for the pairs SBS3-SBS40 and SBS5-SBS92, while the median flatness, calculated through Eq. 2, is 0.76. These six signatures were used to build up the first extraction scenario presented in Table 1.

A second notable group is characterised by a high pairwise cosine similarity among signatures but with a low level of flatness and it includes SBS36, SBS18 and the three signatures SBS10a, SBS10c and SBS10d associated with an altered activity of polymerase (polymerase epsilon exonuclease domain mutations and defective POLD1 proofreading), which were considered for the second extraction scenario. The median pairwise similarity is 0.83 with a maximum equal to 0.91 between SBS36 and SBS18 while the median flatness is 0.34. In the third extraction scenario the synthetic catalogues were generated from the signatures used in the first and the second scenario together (11 signatures). Finally, in the fourth and fifth extraction scenarios, 11 and 20 signatures with a low flatness score were considered, respectively, where each signature has at least another similar one (cosine similarity > 0.8). The complete list of signatures used in each scenario and the list of those signatures with a cosine similarity >0.8 were reported in Supplementary Tables S1 and S2, respectively. In Supplementary Figure S1 we reported the pairwise similarity distribution for each scenario compared with the full set of non-artefactual COSMIC signatures. The first and second scenarios have a very high similarity level as they were built by taking the two largest clusters of the similarity-based cluster map. The others are gradually less similar since the considered number of signatures increases but the number of similar signatures in each cluster decreases.

### Flatness analysis

**FIGURE 3:**
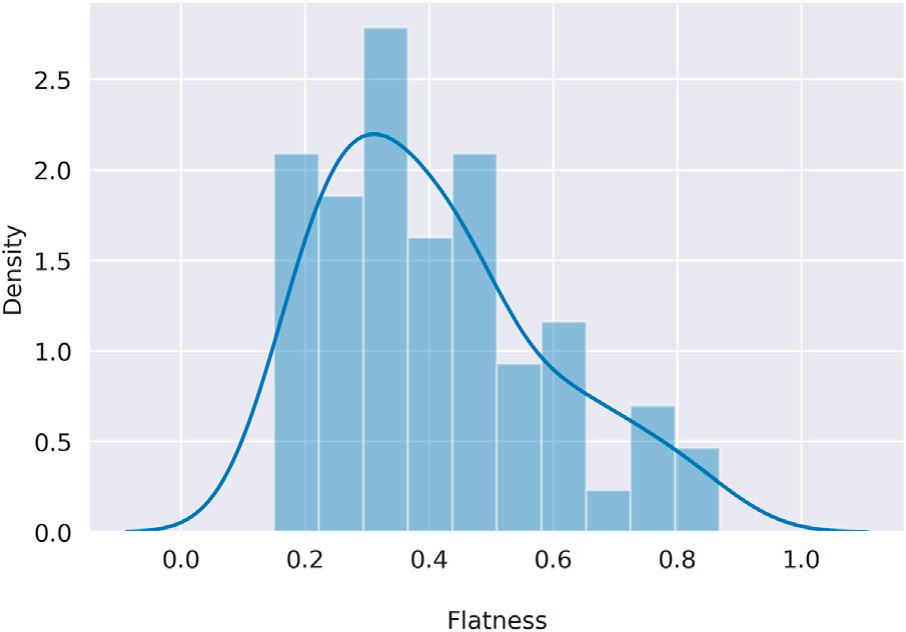
Distribution of the COSMIC flatness. On the *x* axis the flatness defined in Eq. 2, on the *y* axis the density for each flatness level.

To overcome the qualitative description of flatness, in Eq. (2) (Methods section) we defined a simple way to quantitatively assess the flatness of the signatures, being in line with the qualitative description. Indeed, as shown in Supplementary Table S3, the known flat signatures SBS3, SBS40 and SBS5 show the highest degree of flatness, but a similar level to SBS5 can be found for SBS25 and SBS89. In addition, this definition of flatness appears to be well distributed within COSMIC from a minimum of 0.15 (SBS1) to a maximum of 0.87 (SBS3), showing that this metric can emphasise the differences in shape between the various signatures in COSMIC, as shown in Figure 3. As mentioned in the previous section, the extraction scenarios, built to highlight possible issues in the *de novo* extraction process, differ in the number of signatures involved, the pairwise similarity between profiles, and the level of flatness. In Supplementary Figure S2, the flatness distribution for each scenario is shown.

### *De novo* signature extraction

The SigProfilerExtractor performance for each scenario is shown in Table 2. MSE, *C*_*mean*_ and *C*_min_ are reported as their corresponding median values across 10 repetitions, together with their inter-quartile range. When considered separately, signatures involved in scenarios 1 and 2 were almost always correctly extracted at 200 samples (F = 0.9), regardless of the high level of similarity in each group.

**TABLE 2:** *De novo* signature extraction performance. For each simulated scenario, the frequency of runs with all the signatures correctly identified F), the mean square error (MSE) between simulated and reconstructed catalogues, the average *C*_*mean*_ and minimum *C*_*min*_ stability scores of signature clusters are displayed.

Scenario	Number of samples	F	MSE	C_*mean*_	C_*min*_
1	200	0.9	45.59 (45.45, 46.34)	0.88 (0.86–0.89)	0.78 (0.74–0.82)
	500	1	46.64 (46.41, 46.89)	0.93 (0.9–0.94)	0.86 (0.75–0.88)
2	200	0.9	27.75 (27.62, 28.05)	0.84 (0.83–0.86)	0.67 (0.54–0.69)
	500	1	28.11 (27.85, 28.45)	0.87 (0.86–0.89)	0.69 0.65–0.72
3	200	0.0	124.36 (120.66, 127.48)	0.98 (0.97–0.98)	0.96 (0.94–0.97)
	500	0.0	129.08 (126.64, 130.17)	0.99 (0.99–0.99)	0.98 (0.97–0.99)
	1,000	0.0	90.49 (41.68, 127.92)	0.90 (0.81–0.99)	0.80 (0.58–0.98)
	3,000	0.0	41.05 (40.89, 41.28)	0.81 (0.8–0.82)	0.47 (0.41–0.5)
	5,000	0.0	40.18 (38.99, 41.38)	0.81 (0.80–0.82)	0.35 (0.26–0.49)
	10000	0.8	35.58 (35.55, 35.72)	0.81 (0.80–0.81)	0.36 (0.33–0.47)
4	200	0.0	101.93 (85.45, 103.0)	0.88 (0.84, 0.9)	0.76 (0.68–0.81)
	500	0.1	40.38 (38.24, 41.01)	0.82 (0.82–0.83)	0.38 (0.3–0.45)
	1,000	0.9	33.20 (32.81, 33.42)	0.83 (0.81–0.85)	0.52 (0.47–0.58)
	3,000	1	32.58 (32.56, 32.69)	0.86 (0.85–0.87)	0.58 (0.53–0.62)
	5,000	1	32.54 (32.36, 32.69)	0.89 (0.87–0.89)	0.67 (0.6–0.7)
5	1,000	0.0	68.98 (67.01, 70.04)	0.83 (0.81–0.86)	0.49 (0.33–0.59)
	3,000	0.0	37.42 (37.16, 37.68)	0.81 (0.81–0.82)	0.33 (0.26–0.39)
	5,000	0.0	37.01 (36.94, 37.12)	0.81 (0.80–0.82)	0.34 (0.29–0.39)
	10000	0.1	36.63 (36.44, 36.77)	0.81 (0.81–0.82)	0.38 (0.32–0.4)

However, when the extraction was performed by combining these two scenarios (scenario 3), SigProfilerExtractor was never able to identify the correct number of signatures up to a high number of samples (5,000) and only at 10,000 samples it succeeded 80% of the times (F = 0.8). In this scenario it is worth noticing that, as the number of available samples increases, while the MSE decreases and the average stability decreases but remaining relatively high, the minimum stability dramatically decreases. As expected, by further increasing the number of samples, both the mean and minimum stability values rise again. However, given that obtaining 10,000 tumour samples is often unfeasible in practice, this scenario highlights well a limitation of the NMF-based extraction process. Indeed, this scenario is particularly complex since it considers two main subgroups, highly similar internally (median pairwise cosine similarity 0.73 and 0.83, respectively) but one at high and the other one at low flatness score (0.76 and 0.34, respectively, as shown in Table 1). Therefore, this difference in the flatness levels makes the extraction process much more difficult if there is not a very large number of samples. As shown in Supplementary Figure S3, the algorithm starts to differentiate similar signatures inside each of these two groups at 3,000 samples, but still failing at differentiating them well even at 5,000 samples.

On the other hand, considering again 11 signatures but with a lower level of similarity and flatness (scenario 4), the algorithm required at least 1,000 samples to identify the signatures with F = 0.9 (Supplementary Figure S3). Finally, when 20 signatures were considered (scenario 5), the algorithm always failed even at 5,000 samples, and it only succeeded 10% of the times at 10,000 samples. It is worth highlighting that the maximum number considered is significantly higher compared to the 2,780 genomes from PCAWG used to build the gold-standard catalogue of mutational signatures available in COSMIC.

### Archetypal analysis

The application of AA to the COSMIC SBS mutational signature matrix $M\;\in R^{60\times 96}$ , revealed that 29 archetypes $z_{k}\in R^{96}$ (*k* = 1, .., 29) were able to explain the 95% of the variance (Supplementary Figure S4) and that through a combination of them it is possible to reconstruct each COSMIC signature profile with cosine similarity > 0.8 (Supplementary Figure S5).

**FIGURE 4:**
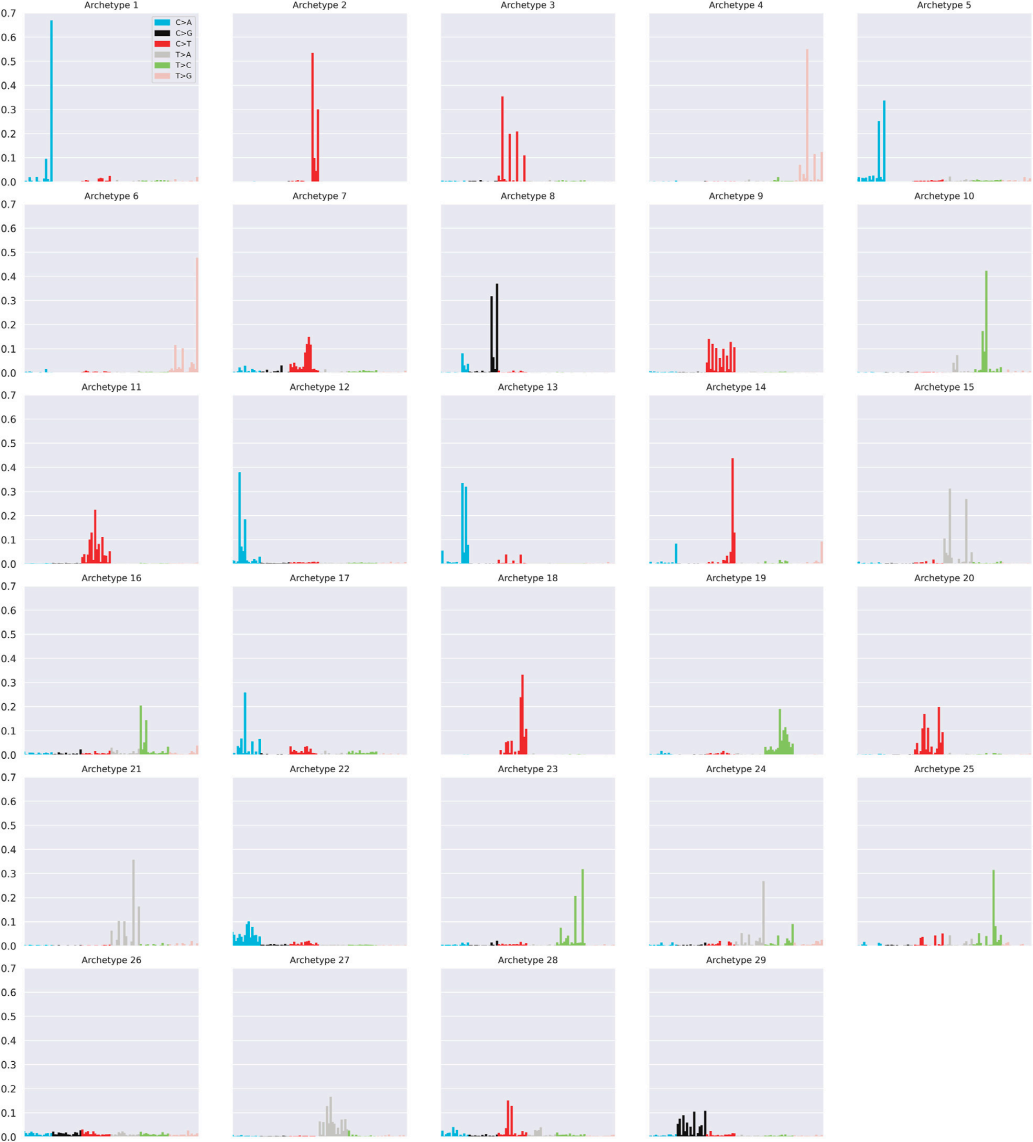
Summary of the 29 archetypal profiles.

The archetypal profiles are summarised in Figure 4. Most of the archetypal profiles coincide almost perfectly with some of the COSMIC signatures. Specifically, 26 out of 29 archetypes correspond to at least one COSMIC signature with cosine similarity of at least 0.97 (Supplementary Figure S6). These results suggest that a subset of signature profiles represents extreme patterns of the catalogue and that a combination of them is capable of reconstructing the entire catalogue with a high level of accuracy. The relationship between signatures and archetypes can be better understood considering the *α* coefficients of Eq (8). In particular, the coefficients *a*_*ik*_ represent the weights that each archetype **z**_**k**_ has in the reconstruction of the *i*−*th* signature **x**_**i**_.

**FIGURE 5:**
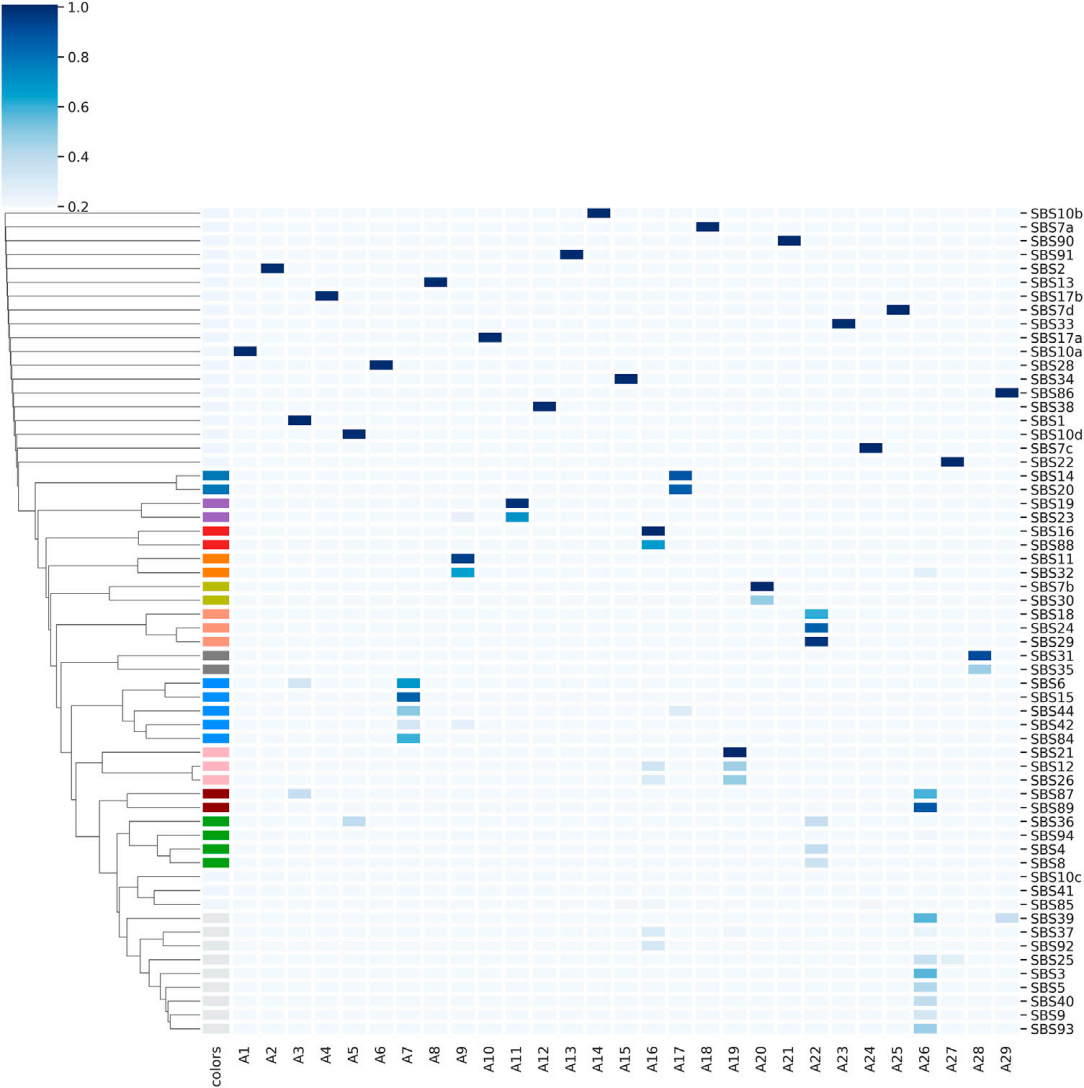
Heatmap highlighting the associations between archetypes and the reconstructed signatures. Different colors highlight groups of reconstructed signatures that are mainly represented by the same archetype. For a better visualisation, *α* coefficients < 0.2, even if used for clustering, were not displayed.

Figure 5 shows the association between the COSMIC signatures and the archetypes through the *α* coefficients. The heatmap was consequently clustered to find those signatures which share a common reconstruction pattern through the archetypes. It can be seen that 19 archetypes reconstruct only one signature, indicating a one-to-one relationship between them. Others were found to contribute in more than one signature at different weights, as well as there are groups of reconstructed signatures that are mainly represented by the same archetype, highlighted by different colours in Figure 5.

Interestingly, AA tends to group similar profiles together fairly well, since the signatures belonging to the same group usually share either the same aetiology or similar biological processes. In Supplementary Figure S7 we further explored the relationship between the mutational signatures and the archetypes by plotting the pairwise cosine similarity distribution of the *α* coefficients for different categories of pairwise cosine similarity between the original signatures. It is possible to clearly observe that, as the pairwise cosine similarity between the signatures increases, the cosine similarity between *α* coefficients increases. This confirms that, while providing a more compact representation of the COSMIC signatures, the archetypal analysis is able to maintain a good consistency with the original profiles.

Table 3 summarises some of the qualitative information that can be extracted from the heatmap, showing the relationships between the reconstructed signatures and the archetype that contributed most to them. Each signature was reported with its aetiology, and whether it had been validated experimentally or by statistical association (i.e. unclear evidence for real signature, as reported in COSMIC).

**TABLE 3:** Aetiological information related to each archetype. For each archetype, the corresponding reconstructed signatures and, when available, their associated aetiologies are reported, indicating the validation studies supporting the biological interpretation.

Color	Main Archetype	Signature	Aetiology	Validation
Blue	A17	SBS14	MMR deficiency + POLE	Experimental Hodel et al. (2020)
		SBS20	MMR deficiency + POLD1	Experimental Meier et al. (2018)
Purple	A11	SBS19	Unknown	-
		SBS23	Unknown	-
Red	A16	SBS16	Unknown	-
		SBS88	Colibactin Exposure	Experimental Pleguezuelos et al. (2020)
Orange	A9	SBS11	Temozolomide treatment	Experimental Kucab et al. (2019)
		SBS32	Azathioprine treatment	Statistical Inman et al. (2018)
Yellow	A20	SBS7b	UV exposure	Experimental Nik-Zainal et al. (2015)
		SBS30	BER deficiency	Experimental Drost et al. (2017)
Salmon	A22	SBS18	Damage by ROS	Experimental Kucab et al. (2019)
		SBS24	Aflatoxin exposure	Experimental Chawanthayatham et al. (2017)
		SBS29	Tobacco chewing	Statistical Alexandrov et al. (2015)
Grey	A28	SBS31	Platinum chemiotherapy	Experimental Boot et al. (2018)
		SBS35	Platinum chemiotherapy	Experimental Boot et al. (2018)
Silver Blue	A7	SBS6	MMR deficiency	Experimental Meier et al. (2018)
		SBS15	MMR deficiency	Experimental Meier et al. (2018)
		SBS44	MMR deficiency	Experimental Drost et al. (2017)
		SBS84	AID activity	Statistical Kasar et al. (2015)
Pink	A19	SBS12	Unknown	-
		SBS21	MMR deficiency	Experimental Meier et al. (2018)
		SBS26	MMR deficiency	Experimental Meier et al. (2018)
Brown	A26	SBS87	Thiopurine treatment	Experimental Li et al. (2020)
		SBS89	Unknown	-
Green	A22	SBS4	Tobacco smoking	Experimental Nik-Zainal et al. (2015)
		SBS8	HR/NER deficiency	Statistical Alexandrov et al. (2013)
		SBS36	BER deficiency (ROS damage)	Experimental Pilati et al. (2017)
		SBS94	Unknown	-
Light grey	A26	SBS3	HR deficiency	Experimental Zámborszky et al. (2017)
		SBS5	Aging/Tobacco/NER deficiency	Statistical Alexandrov et al. (2013)
		SBS9	Polymerase eta hypermutation	Statistical Alexandrov et al. (2013)
		SBS25	Unknown Chemiotherapy	Statistical Alexandrov et al. (2015)
		SBS39	Unknown	-
		SBS40	Unknown	-
		SBS93	Unknown	-

It is possible to observe that seven signatures (SBS6, SBS14, SBS15, SBS20, SBS21, SBS26, and SBS44), experimentally associated with mismatch repair (MMR) deficiency, are divided into three groups: Blue, Silver Blue and Pink. The Blue group includes two signatures associated with the concurrent effect of MMR deficiency and DNA polymerase (POLD1 and POLE), showing a profile mainly polarised on C > A mutations, whereas the Silver Blue and Pink groups have mutational peaks at C > T and T > C, respectively. Thus, although all these seven signatures are involved in MMR deficiency, they probably refer to different types of deficiency in MMR genes. The Silver Blue group, in addition to MMR deficiency associated signatures, also includes SBS84, which is statistically associated with AID activity, found in the immunoglobulin genes and other regions in lymphoid cancers. Although the MMR pathway is generally involved in repairing errors that can occur in DNA during the replication and the recombination, it was found to cooperate with the AID enzyme to generate DNA mutations as part of the antibody diversification process (Martin and Scharff, 2002; Liu and Schatz, 2009; Zanotti and Gearhart, 2016). Thus, MMR pathway and AID mechanism are closely related. In this context, it is very interesting to note that SBS6, although it is implicated in several tumour types, was mainly found in B-Cell Non-Hodgkin Lymphoma samples https://cancer.sanger.ac.uk/signatures/sbs/sbs6/ and that SBS84 is associated with the AID activity in B-Cell Non-Hodgkin Lymphoma. This might suggest that SBS6 relates to an MMR deficiency for genes involved in the development of antibody specificity that cooperates with AID activity. On the other hand, in the Pink group, in addition to the two signatures SBS21 and SBS26 associated with MMR deficiency, there is also SBS12 that was mainly found in liver cancer-related tissue; the high similarity (0.93, Table 2) between the signatures SBS26 and SBS12 and the unknown aetiology could suggest that either SBS12 is also related to MMR deficiency or that these two could actually correspond to the same signature. A cluster formed by these two signatures was also highlighted by Degasperi et al. (2020) by performing an organ-wise mutational signature extraction. Another interesting group is the Grey one, including SBS31 and SBS35, both referring to platinum chemotherapy. The Orange group (SBS11 and SBS32) refers to two treatments as well: Temozolomide, an alkylating agent used as treatment for high-grade brain tumors and melanoma, and Azathioprine, an immunosuppressant. Both these treatments were shown to induce myelosuppression (Connell et al., 1993; Sylvester et al., 2011). The associated signatures are mainly represented by archetype A9, whose profile is characterised by high frequency of C > T mutations. The Yellow group includes SBS7b and SBS30, which are both characterised by C > T mutations and linked with ultraviolet (UV) radiation and base excision repair (BER) deficiency. Recently, it was found that BER increases cellular tolerance to UV independently of nucleic excision repair (Saha et al., 2020). The Salmon group consisted in three signatures (SBS18, SBS24 and SBS29) associated with reactive oxygen species (ROS) damage, aflatoxin exposure and tobacco chewing, respectively. These three signatures are linked together by oxidative stress processes, since it is known that aflatoxin biosynthesis is linked with ROS (Shen et al., 1996; An et al., 2017; Dey and Kang, 2020; Huang et al., 2020), as well as the cytotoxic effects from tobacco chewing are mediated by ROS production (Stich and Anders, 1989; Bagchi et al., 2002). The Green group is composed of SBS4, SBS8, SBS36 and SBS94. These signatures are mainly represented by the archetype A22 as the Salmon group. Indeed SBS29 (Salmon group) associated with tobacco chewing seems to be “complementary” to SBS4 since it was found in some liver and lung cancers where SBS4, related to tobacco smoking, has not been detected. In addition, also SBS36 is associated with BER deficiency including DNA damage due to ROS, as SBS18 in the Salmon group. SBS8 is statistically associated with HR/NER deficiency. Purple, Red and Brown groups include mainly signatures with unknown aetiology and therefore it was not possible to establish qualitative associations between their signatures. Finally, the Light Grey group includes mainly signatures with a high level of flatness (SBS3, SBS5, SBS25 and SBS40) or at least with mutations distributed over all the 96 mutational contexts. Indeed, these signatures are mostly represented by A26, whose profile is in turn homogeneously distributed over all the contexts.

## Discussion

This study investigates the extraction stability issues among the SBS mutational signatures of the most recent version of COSMIC catalogue (3.3). Through a series of simulations considering different scenarios, we showed that high levels of similarity combined with some peculiar (e.g., showing high levels of flatness) signature profiles considerably complicate the *de novo* extraction. Most of the previous studies evaluated stability issues on COSMIC signatures version 2, which includes 30 signatures. However, here we showed that these issues are becoming more critical in the newest version by evaluating 60 non-artefactual signatures. Although SigProfilerExtractor has been proven to be a robust method for signature extraction, even when the number of samples was high (i.e. up to 5,000), it failed in identifying the correct number of signatures and it succeeded 80% of times with 10,000 samples when we simulated a combined set of six similar signatures at high level of flatness and five similar signatures at a lower level of flatness (scenario 3). Similarly, in scenario 5, considering a higher number of latent signatures (20) with at least each signature highly similar (i.e. pairwise cosine similarity >0.8 ) to another one, it always failed up to 5,000 samples and with 10,000 samples it succeeded only 10% of the times.

Although the mutational signatures are not orthogonal by definition, the presence of highly similar signatures, together with the fact that some have a very high level of flatness and there is a lack of an aetiology for many of them, cast some doubts on the real existence of some of these, suggesting that they may be the result of overfitting and hence a mathematical artefact. Several studies already pointed to this issue (Maura et al., 2019; Koh et al., 2021). However, to the best of our knowledge, the most recent assessment of the signature stability observed among COSMIC signatures was performed by Schumann et al. (2019), where they considered the second version of this database, therefore working on half the number of signatures compared to our study and without exploring different scenarios in terms of number of samples, cosine similarity and flat vs non-flat signatures. A limitation of the catalogues used in this work, realised with SigsPack functions, is represented by the random exposure assigned to each latent signature to create the count matrices, subsequently extracted by SigProfilerExtractor. Hence, simulated catalogues may not represent realistic cancer samples. However, this does not affect the technical evaluations of the limitations in the extraction process highlighted by our simulations.

A novelty introduced by this study was the application of AA to investigate whether the information contained in the COSMIC catalogue could be represented more compactly. AA was shown to be an intuitive and straightforward approach to interpret the data like the clustering, but including the flexibility of the matrix factorization (Mørup and Hansen, 2012; Chen et al., 2014). In contrast to the common distance-based approaches, archetypes characterise extremal rather than average properties of the given data and therefore lead to a more compact representation (Abrol and Sharma, 2020). AA is a type of decomposition where convex combinations of extremal points lie on the convex hull of the data and are themselves restricted to being convex combinations of individual observations (Cutler and Breiman, 1994; Mørup and Hansen, 2012). In our study, by applying AA to the COSMIC catalogue, it was possible to identify 29 archetypes able to explain 95% of the variance. Interestingly, it emerged that most of the archetypes correspond almost perfectly (similarity > 0.97) to some signatures and that, through a combination of them, it is possible to reconstruct with a certain degree of accuracy the other signatures of the COSMIC catalogue. As further validation of the reconstruction process, Supplementary Table S4 shows the refitting performance for the simulated catalogues in Scenario 1 with 500 samples, comparing the archetypes to the original COSMIC signatures using MutationalPatterns (Blokzijl et al., 2018). As expected, since the simulated catalogues are generated based on the original signatures, these latter showed high cosine similarity and low mean absolute error (MAE), i.e. 0.967 and 6.2 on average, respectively. However, keeping in mind that the archetypes explain the 95% of the variance in the simulated catalogue, they were able to perform well by achieving average cosine similarity and MAE equal to 0.958 and 9.91, respectively.

However, it is worth highlighting that archetypes do not substitute the COSMIC signatures, but emphasise the importance of considering alternative approaches able to reduce redundant information. These observations, together with the lack of known aetiology and experimental validation for many signatures, suggest the need to reformulate the COSMIC catalogue using representations including sparsity constraints in latent vectors during the extraction procedure without loss of information. In the future, archetypal analysis can be also considered to evaluate sparse representations of signatures not only in the context of single base substitutions but also for other types of variants like copy number variations and structural variants (Heller et al., 2020; Steele et al., 2022).

## Data Availability

The datasets presented in this study can be found in online repositories. The names of the repository/repositories and accession number(s) can be found below: https://github.com/compbiomed-unito/archetypal-analysis-cosmic.
