# Supplementary material for: Unravelling the instability of mutational signatures extraction via archetypal analysis
Source: Front Genet. 2023 Jan 4;13:1049501. doi: 10.3389/fgene.2022.1049501 (PMC9846778; doi:10.3389/fgene.2022.1049501)
Supplement: Supplementary file 1 [file DataSheet1.PDF]

# Supplementary Material

## Supplementary Tables

**Table S1. SBS Signatures used in each extraction scenario**

| Scenario | SBS Signatures |
|---|---|
| 1 | 3-5-25-40-89-92 |
| 2 | 10a-10c-10d-18-36 |
| 3 | 3-5-25-40-89-92-10a-10c-10d-18-36 |
| 4 | 6-10a-10c-10d-12-15-18-24-26-29-36 |
| 5 | 4-6-8-10a-10c-10d-12-15-18-19-23-24-26-29-36-37-39-86-92-94 |

**Table S2. Pairwise cosine similarity ($>$ 0.8) between COSMIC SBS Mutational Signatures**

| Signature 1 | Signature 2 | Cosine Similarity |
|---|---|---|
| SBS26 | SBS12 | 0.93 |
| SBS36 | SBS18 | 0.91 |
| SBS92 | SBS5 | 0.88 |
| SBS40 | SBS3 | 0.88 |
| SBS36 | SBS10d | 0.88 |
| SBS10d | SBS10a | 0.87 |
| SBS15 | SBS6 | 0.86 |
| SBS29 | SBS24 | 0.86 |
| SBS10c | SBS10a | 0.86 |
| SBS10d | SBS10c | 0.86 |
| SBS94 | SBS4 | 0.85 |
| SBS40 | SBS5 | 0.83 |
| SBS29 | SBS18 | 0.83 |
| SBS37 | SBS12 | 0.82 |
| SBS8 | SBS4 | 0.82 |
| SBS23 | SBS19 | 0.81 |
| SBS86 | SBS39 | 0.81 |
| SBS36 | SBS10c | 0.81 |
| SBS89 | SBS3 | 0.81 |

**Table S3. The ten most flat signatures of COSMIC**

| Signature | Flatness |
|---|---|
| SBS3 | 0.87 |
| SBS40 | 0.83 |
| SBS5 | 0.78 |
| SBS89 | 0.75 |
| SBS25 | 0.74 |
| SBS39 | 0.72 |
| SBS94 | 0.64 |
| SBS9 | 0.64 |
| SBS92 | 0.64 |
| SBS8 | 0.64 |

**Table S4. Cosine similarity and mean absolute error (MAE) between the original catalogues and the reconstructed ones, using MutationalPatterns as a refitting tool. The catalogues are those used in Scenario 1 with 500 samples. Performance is reported in terms of median and interquartile range over 10 runs.**

| Profiles | Cosine Similarity | MAE |
|---|---|---|
| Archetypes | 0.958 (0.958-0.959) | 9.91 (9.89-9.92) |
| COSMIC SBS | 0.967 (0.967-0.968) | 6.20 (6.18-6.24) |

## SUPPLEMENTARY FIGURES

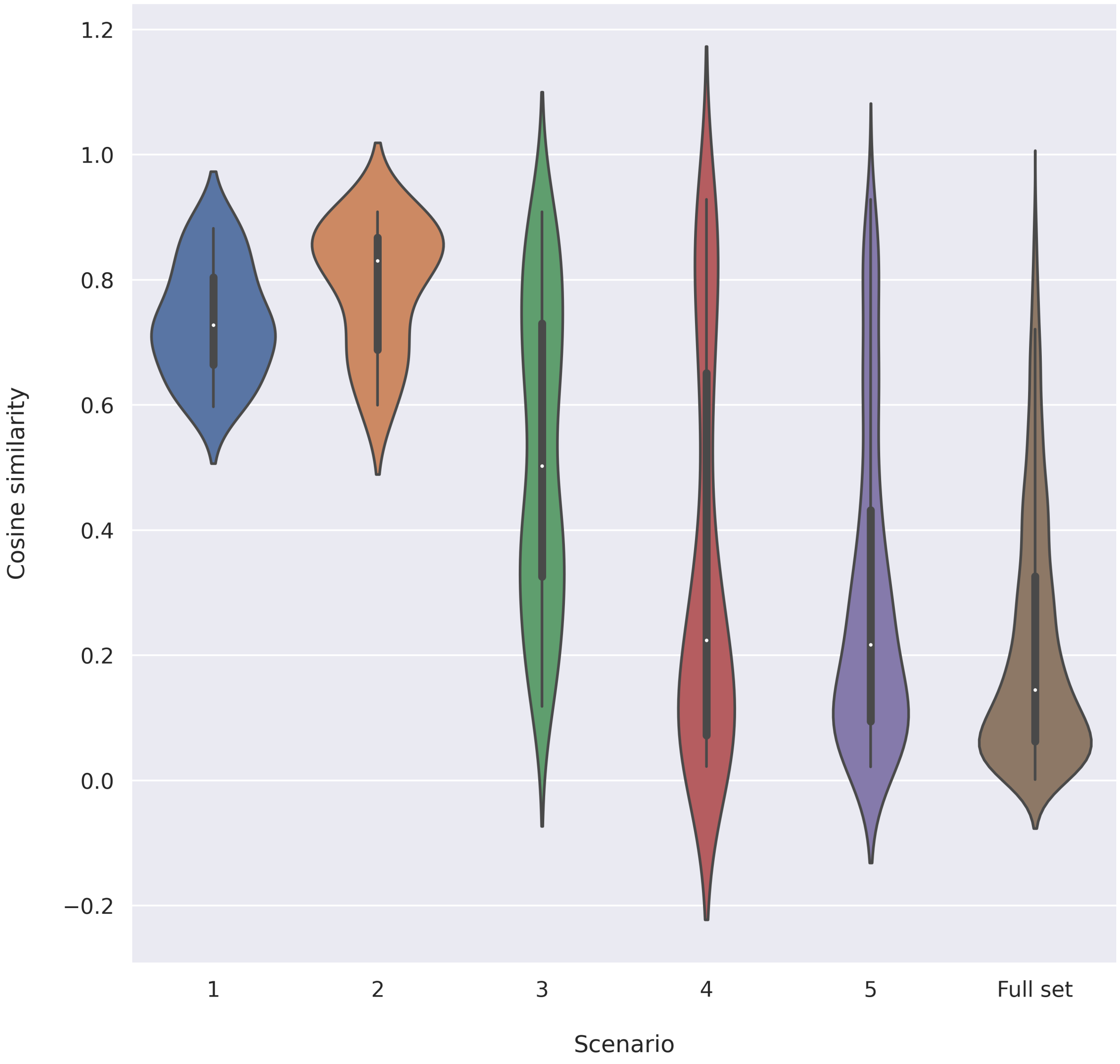


Figure S1: **Cosine similarity distribution for each simulated scenario compared with the full set of non-artefactual COSMIC signatures.**

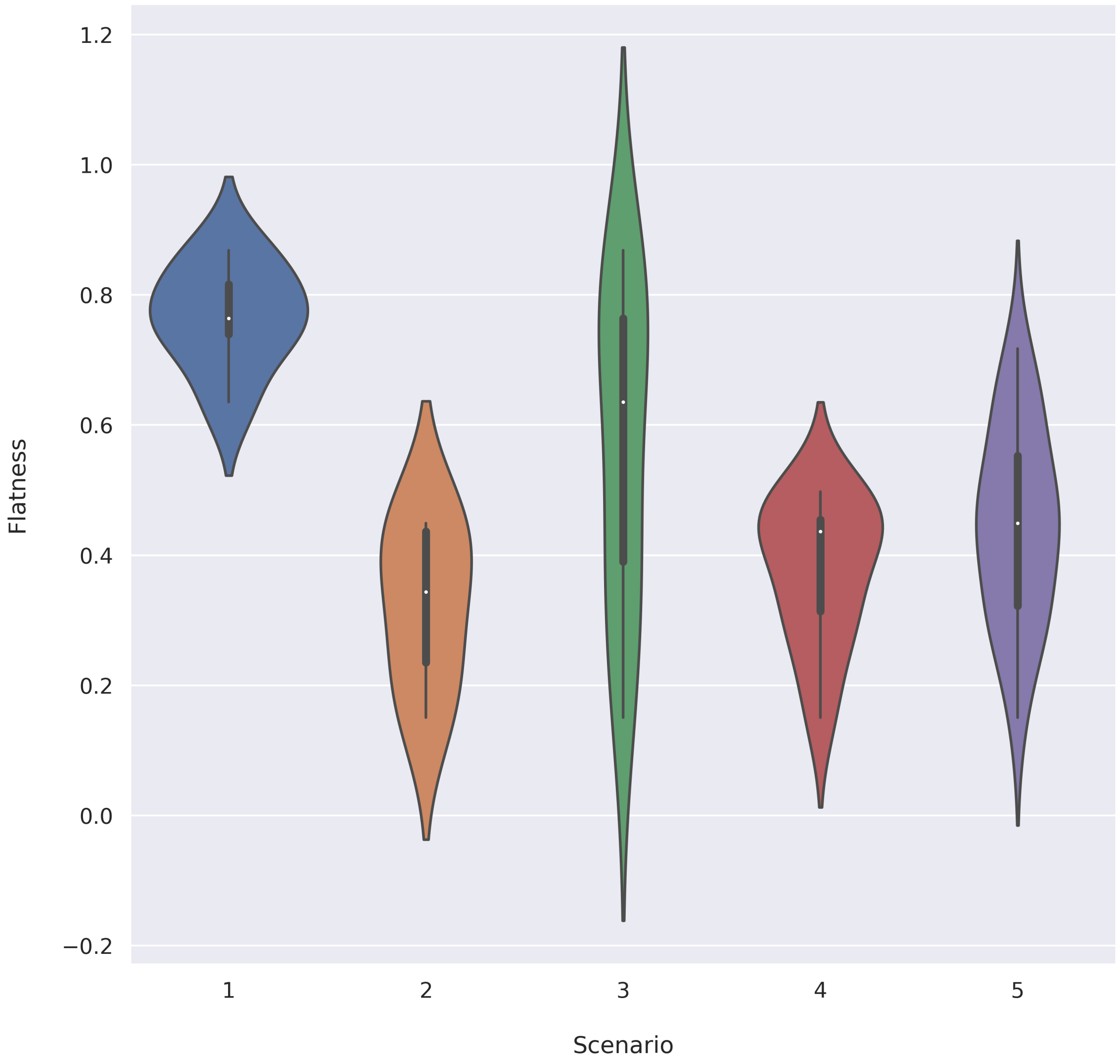


Figure S2: **Flatness distribution for each simulated scenario.**

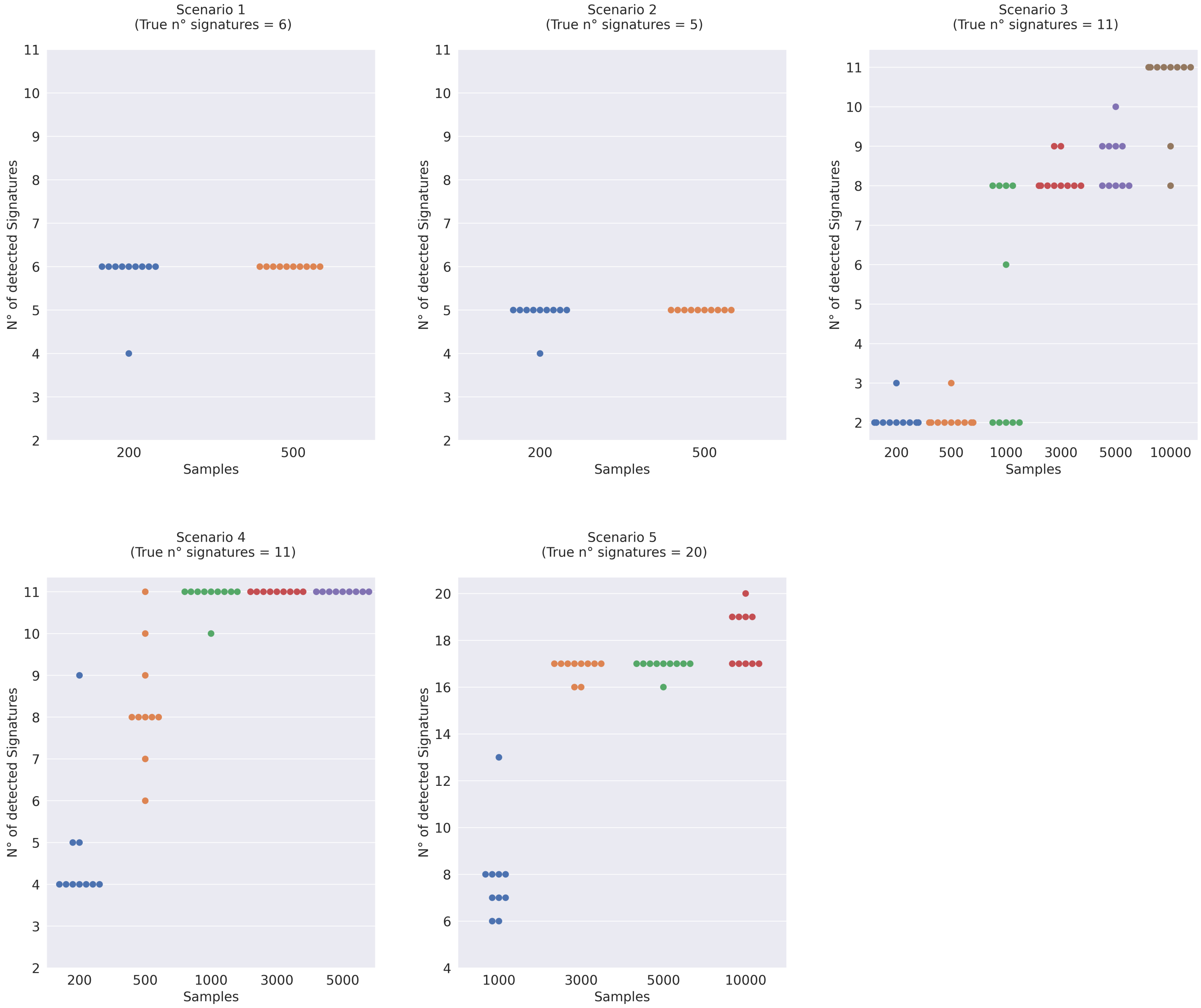


Figure S3: **Number of detected signatures for each simulated scenario.** Each point at a fixed number of samples represents an individual run.

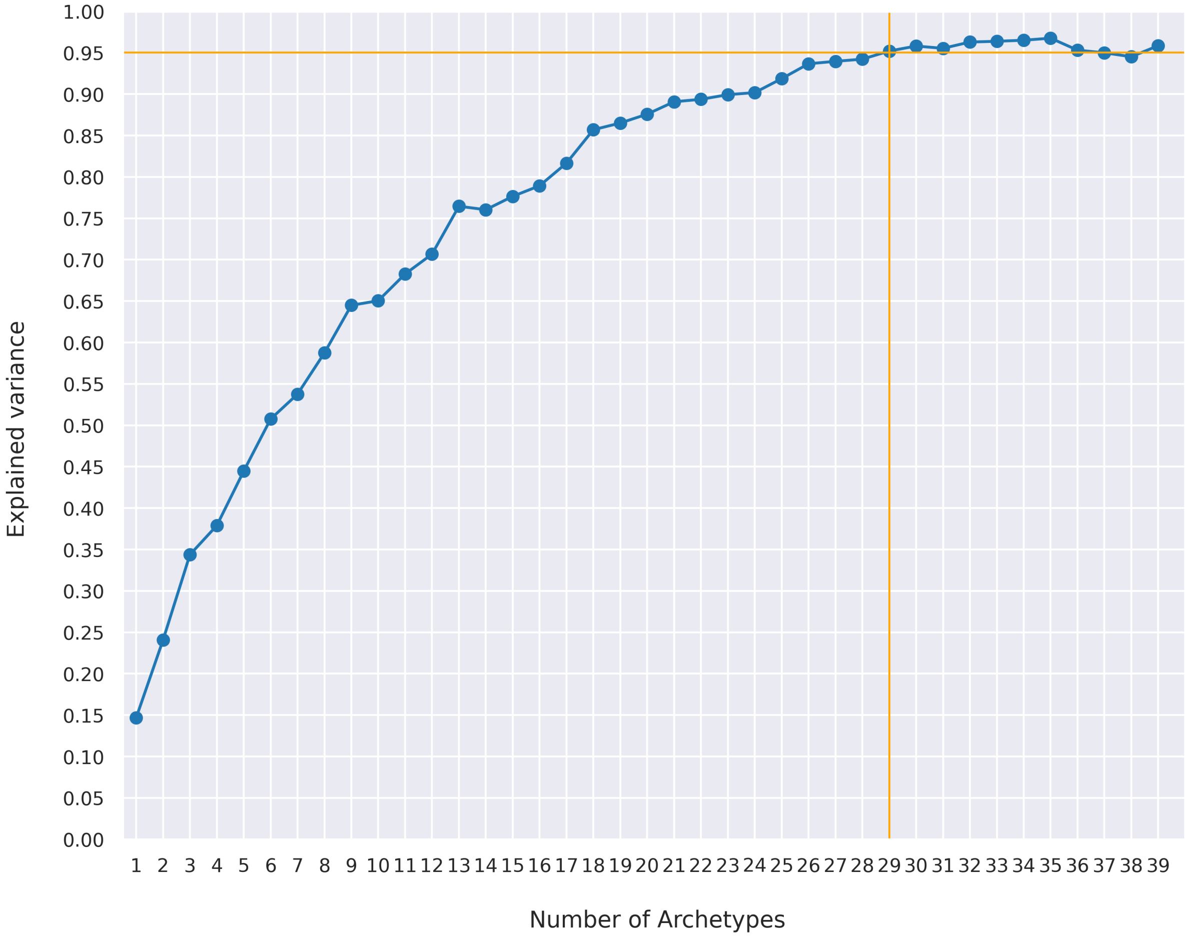


Figure S4: **Explained variance with respect to the number of archetypes.**

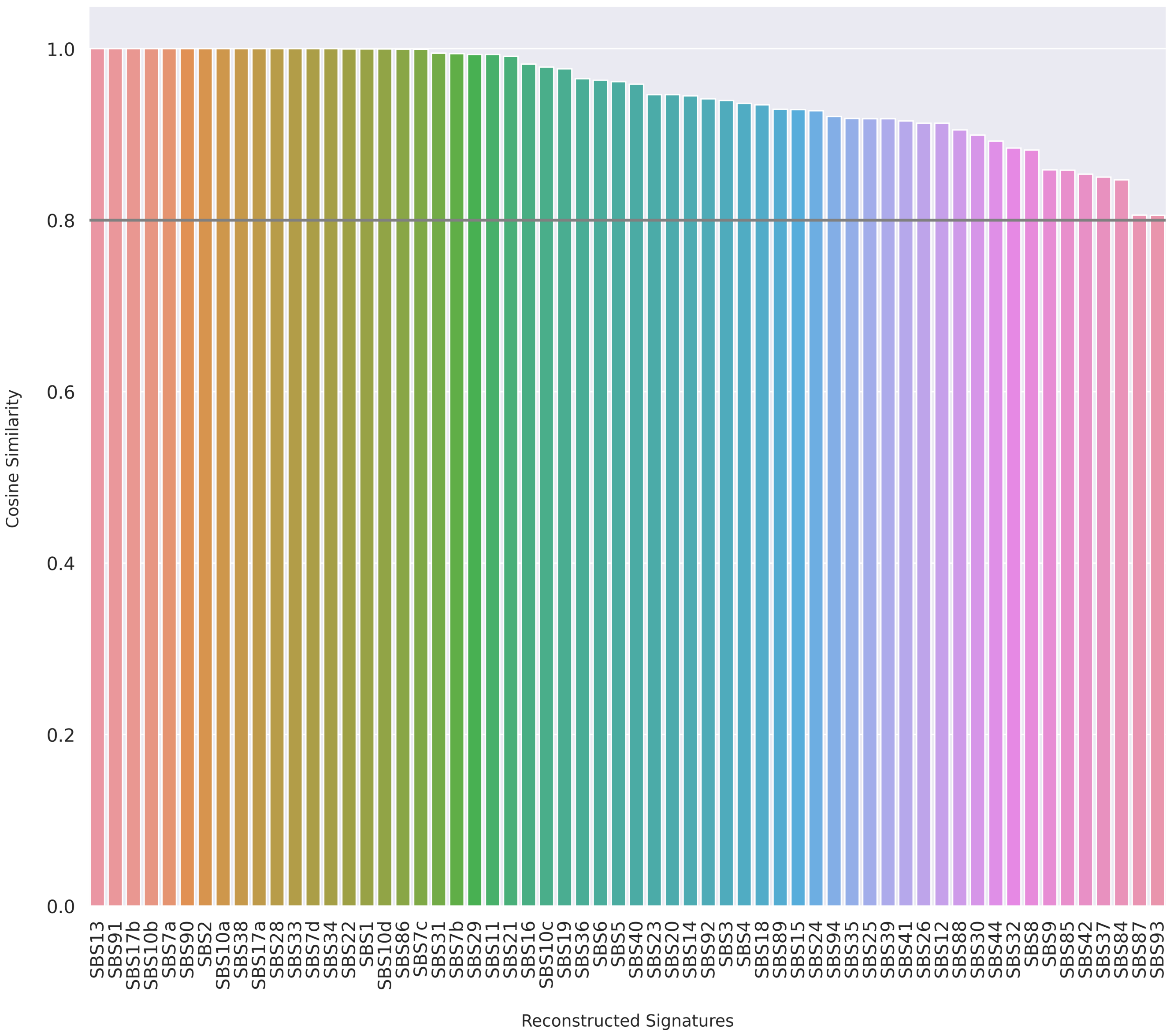


Figure S5: **Cosine similarity between the original COSMIC signatures and the reconstructed ones through the 29 archetypes.**

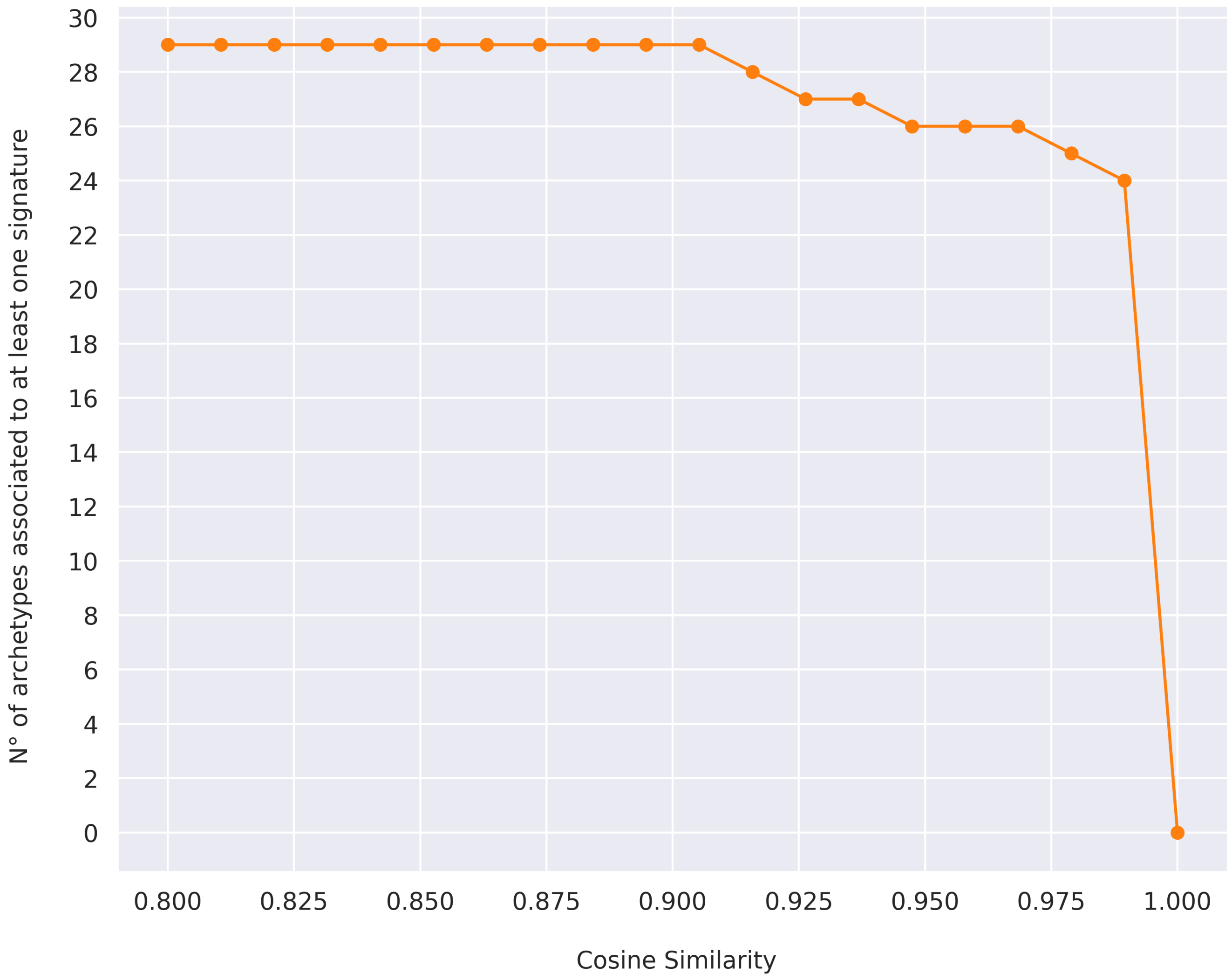


Figure S6: **Relationship between archetypes and signatures at different cosine similarity thresholds.**

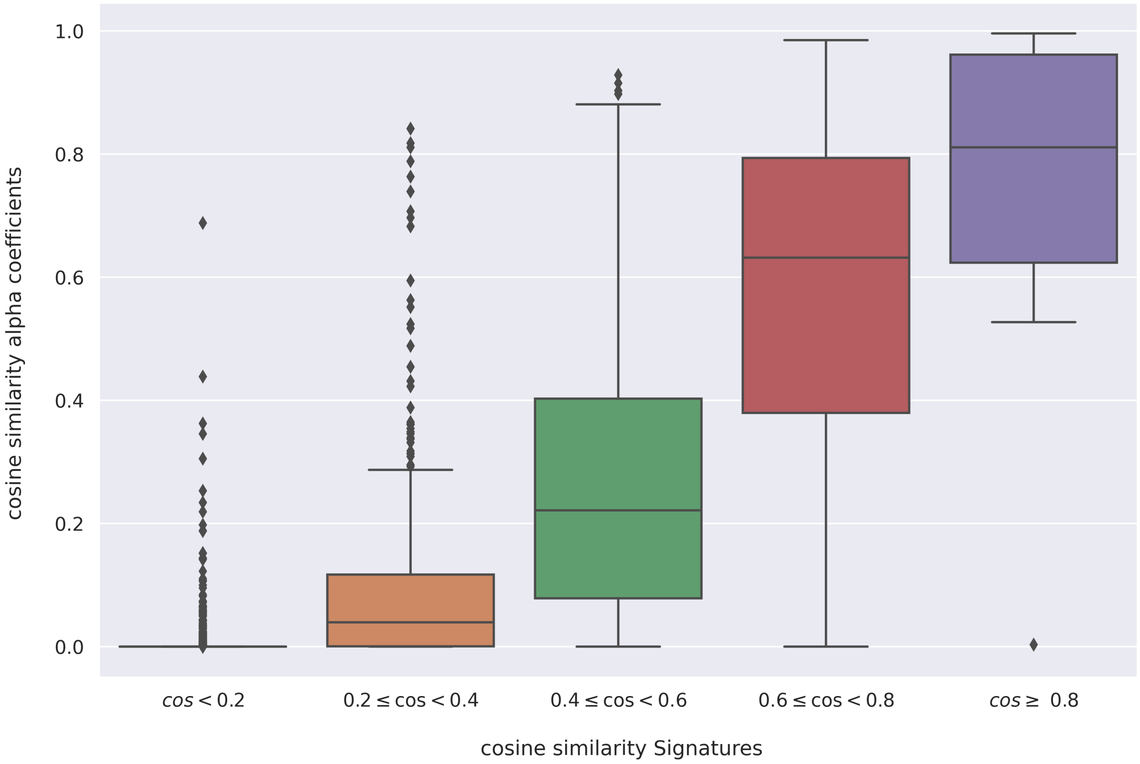


Figure S7: **Pairwise cosine similarity distribution of the alpha coefficients profiles for different levels of pairwise cosine similarity between the original mutational signatures**
